# Supplementary material for: HIVprotI: an integrated web based platform for prediction and design of HIV proteins inhibitors
Source: J Cheminform. 2018 Mar 9;10:12. doi: 10.1186/s13321-018-0266-y (PMC5845081; doi:10.1186/s13321-018-0266-y)
Supplement: Supplementary file 2 — Additional file 2. Source code of HIVProtI web server. [file 13321_2018_266_MOESM2_ESM.zip › HIVprotI_Source-code/hivprotI/weka-3-6-6/doc/weka/gui/beans/Classifier.html]

Classifier


---


|  |  |  |
| --- | --- | --- |
| **Overview** \| **Package** \| **Class** \| **Tree** \| **Deprecated** \| **Index** \| **Help** \| **Weka's home** | |  |
| **PREV CLASS**   **NEXT CLASS** | **FRAMES**    **NO FRAMES**     **All Classes** |  |
| SUMMARY: NESTED \| FIELD \| CONSTR \| METHOD | DETAIL: FIELD \| CONSTR \| METHOD |  |


---


## weka.gui.beans Class Classifier

```
java.lang.Object
  java.awt.Component
      java.awt.Container
          javax.swing.JComponent
              javax.swing.JPanel
                  weka.gui.beans.Classifier
```

**All Implemented Interfaces:**: java.awt.image.ImageObserver, java.awt.MenuContainer, java.io.Serializable, java.util.EventListener, javax.accessibility.Accessible, BeanCommon, EventConstraints, InstanceListener, TestSetListener, TrainingSetListener, UserRequestAcceptor, Visible, WekaWrapper

---

``` public class Classifier extends javax.swing.JPanel implements BeanCommon, Visible, WekaWrapper, EventConstraints, java.io.Serializable, UserRequestAcceptor, TrainingSetListener, TestSetListener, InstanceListener ```

Bean that wraps around weka.classifiers

**Since:**
:   1.0

**Version:**
:   $Revision: 7398 $

**Author:**
:   Mark Hall

**See Also:**: `JPanel`, `BeanCommon`, `Visible`, `WekaWrapper`, `Serializable`, `UserRequestAcceptor`, `TrainingSetListener`, `TestSetListener`, Serialized Form

---

**Nested Class Summary**

**Nested classes/interfaces inherited from class javax.swing.JComponent**

`javax.swing.JComponent.AccessibleJComponent`

**Nested classes/interfaces inherited from class java.awt.Component**

`java.awt.Component.BaselineResizeBehavior`


| **Field Summary** | |
| --- | --- |
| `static java.lang.String` | `FILE_EXTENSION`             the extension for serialized models (binary Java serialization) |

**Fields inherited from class javax.swing.JComponent**

`TOOL_TIP_TEXT_KEY, UNDEFINED_CONDITION, WHEN_ANCESTOR_OF_FOCUSED_COMPONENT, WHEN_FOCUSED, WHEN_IN_FOCUSED_WINDOW`

**Fields inherited from class java.awt.Component**

`BOTTOM_ALIGNMENT, CENTER_ALIGNMENT, LEFT_ALIGNMENT, RIGHT_ALIGNMENT, TOP_ALIGNMENT`

**Fields inherited from interface java.awt.image.ImageObserver**

`ABORT, ALLBITS, ERROR, FRAMEBITS, HEIGHT, PROPERTIES, SOMEBITS, WIDTH`


**Constructor Summary**

`Classifier()`
  
          Creates a new `Classifier` instance.


| **Method Summary** | |
| --- | --- |
| `void` | `acceptInstance(InstanceEvent e)`             Accepts an instance for incremental processing. |
| `void` | `acceptTestSet(TestSetEvent e)`             Accepts a test set for a batch trained classifier |
| `void` | `acceptTrainingSet(TrainingSetEvent e)`             Accepts a training set and builds batch classifier |
| `void` | `addBatchClassifierListener(BatchClassifierListener cl)`             Add a batch classifier listener |
| `void` | `addGraphListener(GraphListener cl)`             Add a graph listener |
| `void` | `addIncrementalClassifierListener(IncrementalClassifierListener cl)`             Add an incremental classifier listener |
| `void` | `addTextListener(TextListener cl)`             Add a text listener |
| `boolean` | `connectionAllowed(java.beans.EventSetDescriptor esd)`             Returns true if, at this time, the object will accept a connection according to the supplied EventSetDescriptor |
| `boolean` | `connectionAllowed(java.lang.String eventName)`             Returns true if, at this time, the object will accept a connection with respect to the named event |
| `void` | `connectionNotification(java.lang.String eventName, java.lang.Object source)`             Notify this object that it has been registered as a listener with a source with respect to the named event |
| `void` | `disconnectionNotification(java.lang.String eventName, java.lang.Object source)`             Notify this object that it has been deregistered as a listener with a source with respect to the supplied event name |
| `java.util.Enumeration` | `enumerateRequests()`             Return an enumeration of requests that can be made by the user |
| `boolean` | `eventGeneratable(java.beans.EventSetDescriptor esd)`             Returns true, if at the current time, the event described by the supplied event descriptor could be generated. |
| `boolean` | `eventGeneratable(java.lang.String eventName)`             Returns true, if at the current time, the named event could be generated. |
| `Classifier` | `getClassifier()`             Get the classifier currently set for this wrapper |
| `Classifier` | `getClassifierTemplate()`             Return the classifier template currently in use. |
| `java.lang.String` | `getCustomName()`             Get the custom (descriptive) name for this bean (if one has been set) |
| `int` | `getExecutionSlots()`             Get the number of execution slots (threads) used to train models. |
| `boolean` | `getUpdateIncrementalClassifier()`             Get whether an incremental classifier will be updated on the incoming instance stream. |
| `BeanVisual` | `getVisual()`             Gets the visual appearance of this wrapper bean |
| `java.lang.Object` | `getWrappedAlgorithm()`             Returns the wrapped classifier |
| `java.lang.String` | `globalInfo()`             Global info (if it exists) for the wrapped classifier |
| `boolean` | `hasIncomingBatchInstances()`             Returns true if this classifier has an incoming connection that is a batch set of instances |
| `boolean` | `hasIncomingStreamInstances()`             Returns true if this classifier has an incoming connection that is an instance stream |
| `boolean` | `isBusy()`             Returns true if. |
| `void` | `loadModel()` |
| `void` | `performRequest(java.lang.String request)`             Perform a particular request |
| `void` | `removeBatchClassifierListener(BatchClassifierListener cl)`             Remove a batch classifier listener |
| `void` | `removeGraphListener(GraphListener cl)`             Remove a graph listener |
| `void` | `removeIncrementalClassifierListener(IncrementalClassifierListener cl)`             Remove an incremental classifier listener |
| `void` | `removeTextListener(TextListener cl)`             Remove a text listener |
| `void` | `saveModel()` |
| `void` | `setClassifierTemplate(Classifier c)`             Set the classifier for this wrapper |
| `void` | `setCustomName(java.lang.String name)`             Set a custom (descriptive) name for this bean |
| `void` | `setExecutionSlots(int slots)`             Set the number of execution slots (threads) to use to train models with. |
| `void` | `setLog(Logger logger)`             Set a logger |
| `void` | `setUpdateIncrementalClassifier(boolean update)`             Set whether an incremental classifier will be updated on the incoming instance stream. |
| `void` | `setVisual(BeanVisual newVisual)`             Sets the visual appearance of this wrapper bean |
| `void` | `setWrappedAlgorithm(java.lang.Object algorithm)`             Sets the algorithm (classifier) for this bean |
| `void` | `stop()`             Stop any classifier action |
| `void` | `useDefaultVisual()`             Use the default visual appearance for this bean |

**Methods inherited from class javax.swing.JPanel**

`getAccessibleContext, getUI, getUIClassID, setUI, updateUI`

**Methods inherited from class javax.swing.JComponent**

`addAncestorListener, addNotify, addVetoableChangeListener, computeVisibleRect, contains, createToolTip, disable, enable, firePropertyChange, firePropertyChange, firePropertyChange, getActionForKeyStroke, getActionMap, getAlignmentX, getAlignmentY, getAncestorListeners, getAutoscrolls, getBaseline, getBaselineResizeBehavior, getBorder, getBounds, getClientProperty, getComponentPopupMenu, getConditionForKeyStroke, getDebugGraphicsOptions, getDefaultLocale, getFontMetrics, getGraphics, getHeight, getInheritsPopupMenu, getInputMap, getInputMap, getInputVerifier, getInsets, getInsets, getListeners, getLocation, getMaximumSize, getMinimumSize, getNextFocusableComponent, getPopupLocation, getPreferredSize, getRegisteredKeyStrokes, getRootPane, getSize, getToolTipLocation, getToolTipText, getToolTipText, getTopLevelAncestor, getTransferHandler, getVerifyInputWhenFocusTarget, getVetoableChangeListeners, getVisibleRect, getWidth, getX, getY, grabFocus, isDoubleBuffered, isLightweightComponent, isManagingFocus, isOpaque, isOptimizedDrawingEnabled, isPaintingForPrint, isPaintingTile, isRequestFocusEnabled, isValidateRoot, paint, paintImmediately, paintImmediately, print, printAll, putClientProperty, registerKeyboardAction, registerKeyboardAction, removeAncestorListener, removeNotify, removeVetoableChangeListener, repaint, repaint, requestDefaultFocus, requestFocus, requestFocus, requestFocusInWindow, resetKeyboardActions, reshape, revalidate, scrollRectToVisible, setActionMap, setAlignmentX, setAlignmentY, setAutoscrolls, setBackground, setBorder, setComponentPopupMenu, setDebugGraphicsOptions, setDefaultLocale, setDoubleBuffered, setEnabled, setFocusTraversalKeys, setFont, setForeground, setInheritsPopupMenu, setInputMap, setInputVerifier, setMaximumSize, setMinimumSize, setNextFocusableComponent, setOpaque, setPreferredSize, setRequestFocusEnabled, setToolTipText, setTransferHandler, setVerifyInputWhenFocusTarget, setVisible, unregisterKeyboardAction, update`

**Methods inherited from class java.awt.Container**

`add, add, add, add, add, addContainerListener, addPropertyChangeListener, addPropertyChangeListener, applyComponentOrientation, areFocusTraversalKeysSet, countComponents, deliverEvent, doLayout, findComponentAt, findComponentAt, getComponent, getComponentAt, getComponentAt, getComponentCount, getComponents, getComponentZOrder, getContainerListeners, getFocusTraversalKeys, getFocusTraversalPolicy, getLayout, getMousePosition, insets, invalidate, isAncestorOf, isFocusCycleRoot, isFocusCycleRoot, isFocusTraversalPolicyProvider, isFocusTraversalPolicySet, layout, list, list, locate, minimumSize, paintComponents, preferredSize, printComponents, remove, remove, removeAll, removeContainerListener, setComponentZOrder, setFocusCycleRoot, setFocusTraversalPolicy, setFocusTraversalPolicyProvider, setLayout, transferFocusBackward, transferFocusDownCycle, validate`

**Methods inherited from class java.awt.Component**

`action, add, addComponentListener, addFocusListener, addHierarchyBoundsListener, addHierarchyListener, addInputMethodListener, addKeyListener, addMouseListener, addMouseMotionListener, addMouseWheelListener, bounds, checkImage, checkImage, contains, createImage, createImage, createVolatileImage, createVolatileImage, dispatchEvent, enable, enableInputMethods, firePropertyChange, firePropertyChange, firePropertyChange, firePropertyChange, firePropertyChange, getBackground, getBounds, getColorModel, getComponentListeners, getComponentOrientation, getCursor, getDropTarget, getFocusCycleRootAncestor, getFocusListeners, getFocusTraversalKeysEnabled, getFont, getForeground, getGraphicsConfiguration, getHierarchyBoundsListeners, getHierarchyListeners, getIgnoreRepaint, getInputContext, getInputMethodListeners, getInputMethodRequests, getKeyListeners, getLocale, getLocation, getLocationOnScreen, getMouseListeners, getMouseMotionListeners, getMousePosition, getMouseWheelListeners, getName, getParent, getPeer, getPropertyChangeListeners, getPropertyChangeListeners, getSize, getToolkit, getTreeLock, gotFocus, handleEvent, hasFocus, hide, imageUpdate, inside, isBackgroundSet, isCursorSet, isDisplayable, isEnabled, isFocusable, isFocusOwner, isFocusTraversable, isFontSet, isForegroundSet, isLightweight, isMaximumSizeSet, isMinimumSizeSet, isPreferredSizeSet, isShowing, isValid, isVisible, keyDown, keyUp, list, list, list, location, lostFocus, mouseDown, mouseDrag, mouseEnter, mouseExit, mouseMove, mouseUp, move, nextFocus, paintAll, postEvent, prepareImage, prepareImage, remove, removeComponentListener, removeFocusListener, removeHierarchyBoundsListener, removeHierarchyListener, removeInputMethodListener, removeKeyListener, removeMouseListener, removeMouseMotionListener, removeMouseWheelListener, removePropertyChangeListener, removePropertyChangeListener, repaint, repaint, repaint, resize, resize, setBounds, setBounds, setComponentOrientation, setCursor, setDropTarget, setFocusable, setFocusTraversalKeysEnabled, setIgnoreRepaint, setLocale, setLocation, setLocation, setName, setSize, setSize, show, show, size, toString, transferFocus, transferFocusUpCycle`

**Methods inherited from class java.lang.Object**

`equals, getClass, hashCode, notify, notifyAll, wait, wait, wait`

**Field Detail**

### FILE\_EXTENSION

```
public static final java.lang.String FILE_EXTENSION
```

:   the extension for serialized models (binary Java serialization)

    **See Also:**: Constant Field Values


**Constructor Detail**

### Classifier

```
public Classifier()
```

:   Creates a new `Classifier` instance.


**Method Detail**

### globalInfo

```
public java.lang.String globalInfo()
```

:   Global info (if it exists) for the wrapped classifier

    :   **Returns:**: the global info

---


### setCustomName

```
public void setCustomName(java.lang.String name)
```

:   Set a custom (descriptive) name for this bean

    :   **Specified by:**: `setCustomName` in interface `BeanCommon`
    :   **Parameters:**: `name` - the name to use

---


### getCustomName

```
public java.lang.String getCustomName()
```

:   Get the custom (descriptive) name for this bean (if one has been set)

    :   **Specified by:**: `getCustomName` in interface `BeanCommon`
    :   **Returns:**: the custom name (or the default name)

---


### getExecutionSlots

```
public int getExecutionSlots()
```

:   Get the number of execution slots (threads) used
    to train models.

    :   **Returns:**: the number of execution slots.

---


### setExecutionSlots

```
public void setExecutionSlots(int slots)
```

:   Set the number of execution slots (threads) to use to
    train models with.

    :   **Parameters:**: `slots` - the number of execution slots to use.

---


### setClassifierTemplate

```
public void setClassifierTemplate(Classifier c)
```

:   Set the classifier for this wrapper

    :   **Parameters:**: `c` - a `weka.classifiers.Classifier` value

---


### getClassifierTemplate

```
public Classifier getClassifierTemplate()
```

:   Return the classifier template currently in use.

    :   **Returns:**: the classifier template currently in use.

---


### hasIncomingStreamInstances

```
public boolean hasIncomingStreamInstances()
```

:   Returns true if this classifier has an incoming connection that is
    an instance stream

    :   **Returns:**: true if has an incoming connection that is an instance stream

---


### hasIncomingBatchInstances

```
public boolean hasIncomingBatchInstances()
```

:   Returns true if this classifier has an incoming connection that is
    a batch set of instances

    :   **Returns:**: a `boolean` value

---


### getClassifier

```
public Classifier getClassifier()
```

:   Get the classifier currently set for this wrapper

    :   **Returns:**: a `weka.classifiers.Classifier` value

---


### setWrappedAlgorithm

```
public void setWrappedAlgorithm(java.lang.Object algorithm)
```

:   Sets the algorithm (classifier) for this bean

    :   **Specified by:**: `setWrappedAlgorithm` in interface `WekaWrapper`
    :   **Parameters:**: `algorithm` - an `Object` value **Throws:**: `java.lang.IllegalArgumentException` - if an error occurs

---


### getWrappedAlgorithm

```
public java.lang.Object getWrappedAlgorithm()
```

:   Returns the wrapped classifier

    :   **Specified by:**: `getWrappedAlgorithm` in interface `WekaWrapper`
    :   **Returns:**: an `Object` value

---


### getUpdateIncrementalClassifier

```
public boolean getUpdateIncrementalClassifier()
```

:   Get whether an incremental classifier will be updated on the
    incoming instance stream.

    :   **Returns:**: true if an incremental classifier is to be updated.

---


### setUpdateIncrementalClassifier

```
public void setUpdateIncrementalClassifier(boolean update)
```

:   Set whether an incremental classifier will be updated on the
    incoming instance stream.

    :   **Parameters:**: `update` - true if an incremental classifier is to be updated.

---


### acceptInstance

```
public void acceptInstance(InstanceEvent e)
```

:   Accepts an instance for incremental processing.

    :   **Specified by:**: `acceptInstance` in interface `InstanceListener`
    :   **Parameters:**: `e` - an `InstanceEvent` value

---


### acceptTrainingSet

```
public void acceptTrainingSet(TrainingSetEvent e)
```

:   Accepts a training set and builds batch classifier

    :   **Specified by:**: `acceptTrainingSet` in interface `TrainingSetListener`
    :   **Parameters:**: `e` - a `TrainingSetEvent` value

---


### acceptTestSet

```
public void acceptTestSet(TestSetEvent e)
```

:   Accepts a test set for a batch trained classifier

    :   **Specified by:**: `acceptTestSet` in interface `TestSetListener`
    :   **Parameters:**: `e` - a `TestSetEvent` value

---


### setVisual

```
public void setVisual(BeanVisual newVisual)
```

:   Sets the visual appearance of this wrapper bean

    :   **Specified by:**: `setVisual` in interface `Visible`
    :   **Parameters:**: `newVisual` - a `BeanVisual` value

---


### getVisual

```
public BeanVisual getVisual()
```

:   Gets the visual appearance of this wrapper bean

    :   **Specified by:**: `getVisual` in interface `Visible`
    :   **Returns:**: a `BeanVisual` value

---


### useDefaultVisual

```
public void useDefaultVisual()
```

:   Use the default visual appearance for this bean

    :   **Specified by:**: `useDefaultVisual` in interface `Visible`

---


### addBatchClassifierListener

```
public void addBatchClassifierListener(BatchClassifierListener cl)
```

:   Add a batch classifier listener

    :   **Parameters:**: `cl` - a `BatchClassifierListener` value

---


### removeBatchClassifierListener

```
public void removeBatchClassifierListener(BatchClassifierListener cl)
```

:   Remove a batch classifier listener

    :   **Parameters:**: `cl` - a `BatchClassifierListener` value

---


### addGraphListener

```
public void addGraphListener(GraphListener cl)
```

:   Add a graph listener

    :   **Parameters:**: `cl` - a `GraphListener` value

---


### removeGraphListener

```
public void removeGraphListener(GraphListener cl)
```

:   Remove a graph listener

    :   **Parameters:**: `cl` - a `GraphListener` value

---


### addTextListener

```
public void addTextListener(TextListener cl)
```

:   Add a text listener

    :   **Parameters:**: `cl` - a `TextListener` value

---


### removeTextListener

```
public void removeTextListener(TextListener cl)
```

:   Remove a text listener

    :   **Parameters:**: `cl` - a `TextListener` value

---


### addIncrementalClassifierListener

```
public void addIncrementalClassifierListener(IncrementalClassifierListener cl)
```

:   Add an incremental classifier listener

    :   **Parameters:**: `cl` - an `IncrementalClassifierListener` value

---


### removeIncrementalClassifierListener

```
public void removeIncrementalClassifierListener(IncrementalClassifierListener cl)
```

:   Remove an incremental classifier listener

    :   **Parameters:**: `cl` - an `IncrementalClassifierListener` value

---


### connectionAllowed

```
public boolean connectionAllowed(java.lang.String eventName)
```

:   Returns true if, at this time,
    the object will accept a connection with respect to the named event

    :   **Specified by:**: `connectionAllowed` in interface `BeanCommon`
    :   **Parameters:**: `eventName` - the event **Returns:**: true if the object will accept a connection

---


### connectionAllowed

```
public boolean connectionAllowed(java.beans.EventSetDescriptor esd)
```

:   Returns true if, at this time,
    the object will accept a connection according to the supplied
    EventSetDescriptor

    :   **Specified by:**: `connectionAllowed` in interface `BeanCommon`
    :   **Parameters:**: `esd` - the EventSetDescriptor **Returns:**: true if the object will accept a connection

---


### connectionNotification

```
public void connectionNotification(java.lang.String eventName,
                                   java.lang.Object source)
```

:   Notify this object that it has been registered as a listener with
    a source with respect to the named event

    :   **Specified by:**: `connectionNotification` in interface `BeanCommon`
    :   **Parameters:**: `eventName` - the event: `source` - the source with which this object has been registered as a listener

---


### disconnectionNotification

```
public void disconnectionNotification(java.lang.String eventName,
                                      java.lang.Object source)
```

:   Notify this object that it has been deregistered as a listener with
    a source with respect to the supplied event name

    :   **Specified by:**: `disconnectionNotification` in interface `BeanCommon`
    :   **Parameters:**: `eventName` - the event: `source` - the source with which this object has been registered as a listener

---


### stop

```
public void stop()
```

:   Stop any classifier action

    :   **Specified by:**: `stop` in interface `BeanCommon`

---


### loadModel

```
public void loadModel()
```

---


### saveModel

```
public void saveModel()
```

---


### setLog

```
public void setLog(Logger logger)
```

:   Set a logger

    :   **Specified by:**: `setLog` in interface `BeanCommon`
    :   **Parameters:**: `logger` - a `Logger` value

---


### enumerateRequests

```
public java.util.Enumeration enumerateRequests()
```

:   Return an enumeration of requests that can be made by the user

    :   **Specified by:**: `enumerateRequests` in interface `UserRequestAcceptor`
    :   **Returns:**: an `Enumeration` value

---


### performRequest

```
public void performRequest(java.lang.String request)
```

:   Perform a particular request

    :   **Specified by:**: `performRequest` in interface `UserRequestAcceptor`
    :   **Parameters:**: `request` - the request to perform **Throws:**: `java.lang.IllegalArgumentException` - if an error occurs

---


### eventGeneratable

```
public boolean eventGeneratable(java.beans.EventSetDescriptor esd)
```

:   Returns true, if at the current time, the event described by the
    supplied event descriptor could be generated.

    :   **Parameters:**: `esd` - an `EventSetDescriptor` value **Returns:**: a `boolean` value

---


### eventGeneratable

```
public boolean eventGeneratable(java.lang.String eventName)
```

:   Returns true, if at the current time, the named event could
    be generated. Assumes that the supplied event name is
    an event that could be generated by this bean

    :   **Specified by:**: `eventGeneratable` in interface `EventConstraints`
    :   **Parameters:**: `eventName` - the name of the event in question **Returns:**: true if the named event could be generated at this point in time

---


### isBusy

```
public boolean isBusy()
```

:   Returns true if. at this time, the bean is busy with some
    (i.e. perhaps a worker thread is performing some calculation).

    :   **Specified by:**: `isBusy` in interface `BeanCommon`
    :   **Returns:**: true if the bean is busy.


---


|  |  |  |
| --- | --- | --- |
| **Overview** \| **Package** \| **Class** \| **Tree** \| **Deprecated** \| **Index** \| **Help** \| **Weka's home** | |  |
| **PREV CLASS**   **NEXT CLASS** | **FRAMES**    **NO FRAMES**     **All Classes** |  |
| SUMMARY: NESTED \| FIELD \| CONSTR \| METHOD | DETAIL: FIELD \| CONSTR \| METHOD |  |


---
